# Effectiveness of programmes and interventions to support optimal breastfeeding among children 0–23 months, South Asia: A scoping review

**DOI:** 10.1111/mcn.12697

**Published:** 2018-11-29

**Authors:** Rukundo K. Benedict, Hope C. Craig, Harriet Torlesse, Rebecca J. Stoltzfus

**Affiliations:** ^1^ Division of Nutritional Sciences Cornell University Ithaca New York USA; ^2^ The DHS Program, ICF International The DHS Program Rockville Maryland USA; ^3^ UNICEF, Regional Office for South Asia Kathmandu Nepal

**Keywords:** continued breastfeeding, early initiation of breastfeeding, exclusive breastfeeding, prelacteal feeding, scoping review, South Asia

## Abstract

Most children in South Asia are breastfed at some point in their lives; however, many are not breastfed optimally, including the early initiation of breastfeeding (EIBF) within 1 hr of birth, avoidance of prelacteal feeds (APF), exclusive breastfeeding (EBF) for 6 months, and continued breastfeeding (CBF) up to 2 years of age or beyond. This review identifies and collates evidence on the effectiveness of interventions to support optimal breastfeeding in five countries in South Asia: Afghanistan, Bangladesh, India, Nepal, and Pakistan. A scoping review was conducted of peer‐reviewed and grey literature. The 31 eligible studies included randomized trials and quasi‐experimental designs that were conducted between 1990 and 2015. Data were collated regarding intervention design, characteristics, and effectiveness to support EIBF, APF, EBF, and CBF. Most studies reported a positive impact on breastfeeding outcomes, including 21/25 studies that examined EIBF, 15/19 studies that examined EBF, and 10/10 studies that examined APF. The only study that examined CBF reported no effect. Education, counselling, and maternal, newborn, and child health initiatives were common intervention types with positive effects on breastfeeding outcomes. Interventions were delivered in health facility, community, and home/family environments. Programmes and interventions that reached women and their families with repeated exposure and beginning during pregnancy were more likely to improve EIBF and EBF outcomes. Interventions with no impact on breastfeeding were characterized by short duration, irregular frequency, inappropriate timing, poor coverage, and targeting.

**Key messages**

- Multiple interventions, including education and counselling, community mobilization, mass media, and maternal and newborn health initiatives, delivered across multiple implementation environments (health facility, community, and home/family), are effective in improving breastfeeding practices in South Asia.
- Evidence from studies that did not report a positive impact on breastfeeding practices indicate that intervention timing, frequency, duration, and targeting influence effectiveness.
- Evidence gaps on the impact of interventions on breastfeeding outcomes were identified for specific countries (Afghanistan, Nepal, and Pakistan), breastfeeding outcomes (avoidance of prelacteal feeds and continued breastfeeding), and implementation environments (policy and workplace).

## 1 INTRODUCTION

Breastfeeding has numerous benefits for survival and lifelong health by protecting against childhood infections and breast cancer, increasing intelligence, and probable reductions in overweight, diabetes, and ovarian cancer (UNICEF, 2014; Victora et al., 2016). The lives of more than 800,000 children and 20,000 women could be saved each year if breastfeeding is scaled up to near universal level (Victora et al., 2016), yet too few children and their mothers benefit from appropriate breastfeeding in all regions of the world (UNICEF, 2016).

Most children in South Asia (96%) are breastfed at some point in their lives; however, the majority of children are not breastfed optimally from birth to 2 years (Dibley et al., 2010; UNICEF, 2016). Trends in breastfeeding practices in the five largest South Asian countries found that over half of children do not benefit from early initiation of breastfeeding (EIBF), despite recent improvements in Bangladesh, India, and Nepal (UNICEF, 2016). Further, there have been recent declines in exclusive breastfeeding (EBF) in several South Asian countries, and continued breastfeeding (CBF) is significantly lower at 2 years than 1 year of age (UNICEF, 2016).

The WHO Global Strategy for Infant and Young Child Feeding recommends a set of interventions to improve breastfeeding (WHO, 2003). Existing evidence suggests that these interventions should be delivered concurrently and in multiple settings, including households, communities, workplaces, and health systems (Haroon, Das, Salam, Imdad, & Bhutta, 2013; Sinha et al., 2015). Despite this, there is substantial heterogeneity in breastfeeding practices within and across countries in South Asia (Benedict, Craig, Torlesse, & Stoltzfus, 2018; UNICEF, 2016), reflecting the varying impact of policy and programme action to protect, promote and support breastfeeding in the region.

Scoping reviews map a broad range of literature and present an overview of evidence on a topic and, unlike systematic reviews, do not evaluate the quality of studies (Arksey & O'Malley, 2005). In this review, we explore and summarize evidence from peer‐reviewed and programme evaluation literature on interventions to improve breastfeeding practices in Afghanistan, Bangladesh, India, Nepal, and Pakistan between 1990 and 2015 to inform research, programming and policy efforts in the region. We highlight areas for future research and note the nuanced factors that influence intervention effectiveness and optimal breastfeeding practices in South Asia.

## 2 METHODS

### 2.1 Study framework

A scoping methodology framework by Arksey and O'Malley was used, including a five‐stage review process: (a) identification of the research objectives, (b) identification of relevant studies, (c) selection of studies, (d) mapping of data, and (e) organization, summary, and reporting of the results (Arksey & O'Malley, 2005).

### 2.2 Definitions

Standard WHO breastfeeding indicator definitions were used for EIBF, EBF, and CBF (WHO, 2017), with some exceptions in order to capture the breadth of evidence on what works to improve breastfeeding practices. WHO defines EIBF as the initiation of breastfeeding within 1 hr of birth; this review also included studies that reported EIBF within 30 min, 3 hr, 5 hr, and 24 hr of birth. EBF in infants aged 0–5 months was the primary focus of this review; however, studies that report EBF for narrower groups within this age range were included, as well. Avoidance of prelacteal feeding (APF) was also included and defined as feeding no food/drink to the infant other than breast milk during the first 3 days of life; studies that assessed intake of prelacteal feeds (PF) were also considered. If a study assessed EBF within 3–4 days of birth, the breastfeeding practice was categorized as APF. CBF was examined at 1 year (12–15 months) and 2 years (20–23 months).

Effectiveness to support breastfeeding was defined as a measured improvement (e.g., proportions or odds ratios) of the breastfeeding practice in the study population.

### 2.3 Literature search

A PICOT search strategy, including elements of the Population, Intervention, Comparison, Outcomes, and Time, was used to identify research articles published between January 1990 and December 2016 from PubMed, Web of Science (CORE Collection), GenderWatch, POPLINE, and Sociological Abstract. Search terms were applied with various Boolean operators for country location, programmatic approach, breastfeeding practice, and date of publication (Figure S1). In addition, grey literature published between was January 1990 and December 2016 on programme evaluations was sourced from experts and organizational websites.

### 2.4 Study selection

Eligibility criteria included study location (Afghanistan, Bangladesh, India, Nepal, Pakistan), study design (individual and cluster randomized controlled trials and quasi‐experimental designs for programme evaluations), year conducted (1990–2015), at least one measured breastfeeding practice of interest (EIBF, APF or PF, EBF, CBF), and full‐text availability in English. Duplicate citations were removed, and titles and abstracts were screened to identify relevant studies. Two researchers independently screened the full texts of all potentially relevant articles to assess eligibility. Any discrepancies between reviewers were resolved by discussion and consultation with a third researcher.

### 2.5 Synthesis of data

Data were synthesized by country location, implementation environment, intervention type, and breastfeeding practice. Synthesis of data was guided by an adapted 2016 UNICEF socio‐ecological framework on breastfeeding (UNICEF, 2016). Two researchers extracted data, and a third researcher reviewed data extraction. Any discrepancies were resolved by discussion among the data extraction team.

The three types of implementation environment were (a) health facility environment, (b) community environment, and (c) home/family environment. Studies that examined health facility‐based interventions or health system factors to improve breastfeeding, such as health worker training, were grouped under health facility environment. Community environment included studies that examined interventions delivered through community platforms, including community‐based peer counselling groups, community mobilization, and mass media campaigns. Community mobilization refers to efforts that encourage community members to promote and support breastfeeding through community health committees, meetings, and leadership engagement. Home and family environment included studies on peer support, education, or counselling delivered through home visits to women and/or their family members. Although policy and workplace environments can also influence a woman's opportunity to breastfeed, there were no eligible studies that examined either of these environments.

A set of four intervention types were identified among the selected studies: education and counselling; community mobilization; mass media; and maternal, newborn child health (MNCH) initiatives. These intervention types are not mutually exclusive, for example, several MNCH initiatives involved education and counselling.

## 3 RESULTS

### 3.1 Characteristics of included studies

After excluding duplicates, 1,157 articles were screened and 31 studies were included (Figure S2). All included studies were peer‐reviewed publications or programme evaluations. The majority of included studies were conducted in India (*n* = 14) and Bangladesh (*n* = 11). Fewer studies took place in Pakistan (*n* = 4) and Nepal (*n* = 2), and no eligible studies were conducted in Afghanistan. In total, 22 individual or cluster randomized controlled trials and nine quasi‐experimental programme evaluations were included (Table 1). The breastfeeding practices most often reported were EIBF (*n* = 25 in Bangladesh, India, Nepal, and Pakistan) and EBF (*n* = 19 in Bangladesh, India, and Pakistan). Fewer studies reported APF or PF (*n* = 10 in Bangladesh, India, and Pakistan) or CBF (*n* = 1 in India only). Several studies reported multiple breastfeeding practices. In some cases, effectiveness was reported for one breastfeeding practice, but no significant effect was reported for other breastfeeding practices (Carvalho et al., 2014; More et al., 2012; Sikander et al., 2015; Talukder et al., 2016; Vir et al., 2014).

**Table 1:** Summary of included studies (*N* = 31)

Source, location, and study design	Target groups	Intervention description including implementation environment, intervention type and intervention group (IG), and comparison group (CG)	Results^a^ (IG vs. CG)
Agrawal et al. (2012) India Quasi‐experimental (no control group)	Health workers (HWs), community health workers (CHWs); pregnant women.	Community: Education and counselling IG: Auxiliary nurse midwives (ANM) and Anganwadi workers (AWW) trained on the promotion of EIBF during antenatal care (ANC) and counselling and problem solving skills. CG: Not applicable.	EIBF proportion: Higher among infants of women who receive care from AWW with better knowledge than poorer knowledge: 49% vs. 33%; OR 1.97 (95% CI [1.55, 2.49]), *P* < 0.001. Higher among infants of women receive care from ANM with better knowledge than poorer knowledge: 42% vs. 29%, OR 1.62 (95% CI [1.25, 2.09]); *P* < 0.001.
Ahmed et al. (2011) Bangladesh Cluster randomized controlled trial (RCT)	Community workers (CW) and supervisors; Mothers/newborns.	Community: MNCH initiatives IG: Community workers and supervisors were trained on community‐based KMC, including skin‐to‐skin contact (SSC), and the promotion of essential newborn care. CG: Community workers and supervisors did not receive community‐based KMC training.	EBF proportion (first 2 days of life): Higher in IG than CG: 6.1% vs. 3.2%, *P* < 0.001. Highest in infants given skin to skin contact for at least 7 hr (17.6%).
Akter et al. (2012) Bangladesh Quasi‐experimental (nonequivalent control group)	Pregnant women in third trimester.	Health facility: Education and counselling IG: Groups of 6–8 women in last trimester of pregnancy were given 4 counselling sessions on EIBF, APF, and EBF by researchers. CG: Pregnant women received routine antenatal care visits.	EIBF proportion: Higher in IG than CG: 75.4% vs. 34.5%, *P* = 0.001. APF proportion: Higher in IG than CG: 61.4% vs. 32.8%, *P* = 0.002. EBF proportion (at 1 month): Higher in IG than CG: 64.9% vs. 37.9%, *P* = 0.003.
Arifeen et al. (2009) Bangladesh Cluster RCT	HW and community members; Women during pregnancy and 6 months postpartum.	Health facility: MNCH initiatives Community: MNCH initiatives IG: Implementation of the Integrated Management of Neonatal and Childhood Illnesses (IMNCI) including health worker training and health system strengthening. In addition, support to community activities (e.g., training of village practitioners on sick child care and support to imams to convey key messages). CG: Routine health services.	EBF proportion: Increased by 19% in IG and 9% in CG between baseline and end line (difference in differences of 10 pp, 95% CI [2.65, 17.62]).
Balakrishnan et al. (2016) India Quasi‐experimental study (nonequivalent control group design)	HW and CHW; Women during pregnancy and 6 months postpartum.	Community: Education and counselling IG: AWW, ANW, accredited social health activists (ASHA) and lady health supervisors (LHS) were trained on the provision of maternal and child health care and on the utilization of a mobile health (mhealth) technology to support the continuum of maternal and child care services. Mhealth application included a home visit planner, built‐in scheduler, checklists and videos to help frontline workers perform activities, improve interpersonal communication, and collect data. CG: Routine health services.	EIBF proportion: Higher in IG than CG: 98% (95% CI [80.2, 98.7]) vs. 73%
Bhandari et al. (2003) India Cluster RCT	HW and CHW; Women during pregnancy and 6 months postpartum.	Community: Education and counselling; community mobilization. IG: AWW, ANM and traditional birth attendants (TBA) trained on individual and group counselling using the IMNCI training manual on breastfeeding. Series of job aids on breastfeeding were developed for HW and CHW, and handouts for women. Community meetings, road shows, nutrition fairs, school debates and promotion banners also took place in intervention areas. CG: Routine health services.	APF proportion: Higher in IG than CG: 31% vs. 75%; OR 0.15 (95% CI [0.09, 0.24]), *P* < 0.001. EIBF proportion (within 3 h of birth): Higher in IG than CG: 50% vs. 24%; OR 3.1 (95% CI [2.05, 4.69]), *P* < 0.001. EBF proportion: Higher in IG than CG at 3 months: 79% vs. 48%; OR 4.0 (95% CI [3.01, 5.38]), *P* < 0.001. Higher in IG than CG at 4 months: 69% vs. 12%; OR 16.1 (95% CI [11.2, 23.2]), *P* < 0.001. Higher in IG than CG at 5 months: 49% vs. 9%; OR 14.8 (95% CI [7.2, 30.6]), *P* < 0.001. Higher in IG than CG at 6 months: 42% vs.4%; OR 17.6 (95% CI [6.6, 47.2], *P* < 0.001.
Bhutta et al. (2008) Pakistan Cluster RCT	CHW; Women during pregnancy and at delivery.	Community: Community mobilization Home/family: Education and counselling IG: Standard training of lady health workers (LHW) plus an extra day of training every 3 months (total of 6 extra days) on home‐based newborn care. Included additional curriculum on the promotion of early breastfeeding and avoidance of prelacteal feeds, and training in group counselling and communication strategies. Encouraged LHWs to visit mothers twice during pregnancy. Intervention also included development of community health committees, and basic training and linkage of TBAs with LHWs. CG: Standard LHW training.	EIBF proportion: Higher in IG than CG: 66.1% vs. 21.1% EBF proportion (for first 4 months): Higher in IG than CG: 48.1% vs. 31.2%.
Carvalho, Thacker, Gupta, and Salomon (2014) India Quasi‐experimental (nonequivalent control group design)	Pregnant women and CHW.	Health facility: MNCH initiatives IG: Conditional cash transfers to increase demand for facility based delivery and reproductive health services, including counselling on breastfeeding, provided by the Janani Suraksha Yojana (JSY) programme. CG: National level child health outcomes.	EIBF proportion: Intervention had positive effect of 6.8 pp (95% CI [5.3, 8.3]). EBF proportion (for 6 months): Intervention had non‐significant effect of 1.0 pp (95% CI [0.6, 6.9]).
Fottrell et al. (2013) Bangladesh Cluster RCT	Women's groups; women during pregnancy and at delivery.	Community: Education and counselling IG: Formation of women's groups that involved participatory learning on maternal and neonatal health including breastfeeding. Groups met monthly, participants included TBAs, health service providers, teachers, community leaders and 3% of attendees were men. CG: Routine health services.	EIBF proportion: Odds higher in IG than CG: OR 1.16 (95% CI [1.05, 1.28]). EBF proportion (for at least 6 weeks): Odds higher in IG than CG: OR 1.05 (95% CI [1.00, 1.11]).
Gavhane, Eklare, and Mohamm (2016) India Individual RCT	Mothers with very low birth weight infants (<1,500 g).	Health facility: MNCH initiatives IG: Counselling of mothers KMC and skin‐to‐skin contact for a minimum of 8 hr per day. CG: Conventional medical care.	Breastfeeding proportion at 6 months (exclusive or partial): Similar in IG and CG: 72.7% vs. 72.3%; OR 0.98 (95% CI [0.39, 2.46]), *P* = 0.97.
Haider, Ashworth, Kabir, and Huttly (2000) Bangladesh Cluster RCT	Pregnant women (and their family members).	Home/family: Education and counselling IG: Home‐based peer counselling programme to promote early and exclusive breastfeeding for 6 months (15 visits lasting 20–40 min between third trimester of pregnancy and 6 months after delivery). Counselling was delivered by trained peer counsellors WHO were trained for 10 days using WHO/UNICEF counselling course. CG: No peer counsellor.	EIBF proportion: Higher in IG than CG: 64% vs. 15%, *P* < 0.001. PF proportion: Lower in IG than CG: 31% vs. 89%, *P* < 0.001. EBF proportion (at 5 months): Higher in IG than CG: 70% vs. 6%, *P* < 0.001.
Jahan et al. (2014) Bangladesh Individual RCT	Pregnant women in third trimester and their family members.	Health facility: Education and counselling IG: 1‐hr nutrition education lesson provided to groups of 6–8 pregnant women and any accompanying family members once a month for 3 months in outpatient clinics. Topics included education on early and exclusive breastfeeding. Counsellors were investigators that received 3 weeks of training on areas including nutrition education and behavioural motivation of pregnant women. CG: Routine health services.	EIBF proportion: Higher in IG than CG: 86% vs. 56.7%, *P* = 0.001. EBF proportion (after 1 month): Higher in IG than CG: 84% vs. 69.3%, *P* = 0.003.
Jha, Kumar, Yadav, Singh, and Niraula (2006) Nepal Individual RCT	Families with child aged <5 years.	Home/family: Education and counselling IG: Home‐based intervention delivered through the family health exercise (FHE) programme. Medical students visited families once in a fortnight for 6 months and met with the head of the family, and other members, for 3 hr. Students were accompanied by faculty members, nutritionist, and a social scientist to each village. Health education included information about hygiene, sanitary practices, and mother and child care. CG: Families adjacent to intervention families, where no health education was given.	EIBF proportion (within 3 hr of birth): Similar in IG and CG: 55.6% vs. 31.3%, *P* = 0.219.
Khan et al. (2016) Bangladesh Individual RCT	Women during pregnancy (third trimester) and 6 months postpartum.	Home/family: Education and counselling IG: Eight home‐based breastfeeding counselling sessions between third trimester and 6 months, after birth in addition to randomized food or micronutrient supplements. Counselling was provided by women from the local community, WHO were trained using a 40‐hr WHO and UNICEF breastfeeding counselling course. CG: Routine health messages during ANC visits only.	EBF proportion: Higher in IG than CG at 4 months: 69.0% (95% CI [66.1, 71.9]) vs. 46.6% (95% CI: [41.8, 50.4]). Higher in IG than CG at 6 months: 15.3% (95% CI [10.4, 20.1]) vs. 6.4% (95% CI [1.3, 11.5]).
Khanal, Zhang, and Khanal (2009) Nepal Cluster RCT	Married women of reproductive age.	Community: MNCH initiatives IG: Community‐based programme to manage neonatal infections mobilized existing female community health volunteers (FCHV) to provide antenatal counselling, essential newborn care education, and basic management of sick newborns. CG: Non‐intervention areas.	EIBF proportion: Increased from baseline to follow‐up in IG: 29.0% vs. 43.5%, *P* < 0.001. Proportion in CG was 43.0% at follow‐up (no baseline data).
Kumar et al. (2008) India Cluster RCT	Women during pregnancy and postpartum and their families.	Community: MNCH initiatives Home/family: MNCH initiatives IG: CHW delivered two types of preventive packages: Group A received essential newborn care, including skin‐to‐skin contact and breastfeeding promotion; Group B received the same package plus use of a liquid crystal hypothermal indicator. CHWs delivered the packages in collective meetings and two antenatal and 2 postnatal household visits. CG: Routine health services.	EIBF proportion: Higher in IG than CG: A: 70.6%, B: 67.6%, CG: 15.5%, *P* < 0.0001. PF proportion: Lower in IGs than CG: A: 38.4%, B: 33.5%, CG: 79.9%, *P* < 0.0001
Mahmood, Jamal, and Khan (2011) Pakistan Individual RCT	Healthy, full‐term mothers anticipating normal delivery with intention to EBF for at least 1 month.	Health facility: MNCH initiatives IG: Promotion and support of early skin‐to‐skin contact: Mothers were supported by health workers to give their infants skin‐to‐skin contact for at least 45 min and when the infant had taken the first feed. CG: Conventional care in which infants were not given SSC.	Mean time to initiate first breastfeed: Lower in IG than CG: 41 min vs. 102 min, *P* < 0.001.
Mazumder et al. (2014) India Cluster RCT	CHW, physicians; Breastfeeding women.	Health facility: MNCH initiatives Home/family: MNCH initiatives IG: Intervention comprised three components: CHW training to conduct home visits and counsel mothers on optimal newborn care, including breastfeeding; training of CHW and physicians on IMNCI case management skills; health system strengthening through improved supervision of CHW. CG: Routine health services.	EBF higher in IG than CG: 25.0% vs. 11.6%; RR: 3.19 (95% CI [2.67, 3.81]). CBF proportion (at 1 year): No significant effect between IG and CG: 85.5% vs. 83.2%; RR: 1.02 (95% CI [1.00, 1.04]).
Memon, Khan, Soofi, Baig, and Bhutta (2015) Pakistan Quasi‐experimental (before and after with control)	LHW and CHW; Pregnant women (and some family members).	Health facility: MNCH initiatives Home/family: MNCH initiatives IG: LHW and CHW received training on IMCI‐based training package. Intervention package was implemented by LHW and CHW through monthly household visits, one‐to‐one counselling sessions, video sessions, and group education in communities. Promoted perinatal and newborn care including EIBF. CG: Routine health services.	EIBF proportion: IG showed significant increase from preintervention to postintervention, 42% vs. 55%, *P* < 0.001. No significant change in CG.
Menon et al. (2016) Bangladesh Cluster RCT	CHW; Women during pregnancy and 6 months postpartum.	Community: Community mobilization; mass media. Home/family: Education and counselling IG: Intensive implementation of the Alive & Thrive initiative including an at‐scale behaviour change programme consisting of intensified interpersonal communication on breastfeeding through monthly home visits; mass media campaign; sensitization of community leaders and community mobilization; and policy advocacy to create a supportive environment for optimal breastfeeding practices. CG: Non‐intensive package consisting of standard nutrition counselling on breastfeeding and less‐intensive mass media campaign, community mobilization and policy advocacy.	EIBF proportion: Increase between baseline and end line greater in IG (64% to 94%) than CG (63% to 77%). Adjusted difference in differences estimate (DDE) 17 pp, (95% CI [3, 31]), *P* = 0.021. PF proportion: Decrease between baseline and end line greater in IG (51% to 10%) than CG (24% to 32%). Adjusted DDE −49 pp, (95% CI [−66, −33]), *P* < 0.001. EBF proportion: Increase between baseline and end line greater in IG (48% to 88%) than CG (51% to 54%). Adjusted DDE 36 pp, (95% CI [21, 51]), *P* < 0.001.
More et al. (2012) India Cluster RCT	Women of reproductive age.	Community: Community mobilization; education and counselling IG: One full‐time, trained facilitator (*sakhi*) was assigned to set up 10 women's groups for women of reproductive age. The groups met fortnightly over 6 months and used “appreciative inquiry” techniques (positive reinforcement) to discuss problems in pregnancy, delivery postpartum and newborn, and ways to address them. Each *sakhi* met weekly with other *sakhis* and her supervisor. CG: No intervention received.	EIBF proportion (within 24 hr of birth): Similar in IG and CG: 82.8% vs. 82.4%; OR: 1.10 (95% CI [0.89, 1.36]). EBF proportion (for at least 4 weeks): Similar in IG and CG: 70.5% vs. 66.7%; OR: 1.21 (95% CI [0.95, 1.54]).
Prasad & Costello (1995) India Quasi experimental (nonequivalent control group)	Hospital medical staff; Mothers with spontaneous normal delivery, and hospital staff.	Health facility: Education and counselling IG: Health education training of hospital staff (hospital administrators, doctors, ward sister, nurses, and midwives) individually or in small groups in at least 5 contacts. Staff were trained on the benefits and feasibility of early breastfeeding, and the dangers of prelacteal feeds, together with instructions on explaining this information to mothers. Midwives and nurses were trained to motivate, persuade, and help mothers to EIBF. CG: Pretraining intervention.	At 6 months following the intervention 36% of women reported receiving education vs. 64% that did not report receiving education. EIBF proportion (within 12 hr): At 6 months following the intervention, higher among mothers who received education (78%) vs. women that received no education 17% (*P* < 0.001). APF proportion: At 6 months following the intervention, higher among mothers who received education (58%) vs. women that received no education 3% (*P* < 0.001).
Rahman et al. (2016) Bangladesh Quasi‐experimental (before and after with control)	CHW; Pregnant women, children under 5 years and their mothers.	Home/family: MNCH initiatives IG: Frontline community health workers, including Shashtho Shebikas and Shastho Kormis received training on the Improving Maternal, Neonatal, and Child Survival (IMNCS). Intervention package delivered to pregnant women and children under five years and their caregivers, and included promotion of EIBF and EBF. CG: Received only “Essential Health Care Programme” (without breastfeeding promotion).	EIBF proportion: Increase between pre and post intervention greater in IG (78% to 90%) than CG (83% to 86%). DDE 8.3 pp, (95% CI [2.6, 13.9]), *P* = 0.0004.
Sikander et al. (2015) Pakistan Cluster RCT	LHW; Women during pregnancy (third trimester) and 6 months postpartum and their families.	Home/family: Education and counselling IG: LHWs trained over 2 days on cognitive‐behavioural counselling and participatory approaches to research. Pregnant and breastfeeding women and their families then received 7 sessions of cognitive‐behavioural counselling on breastfeeding at home, between third trimester and 6 months postpartum. CG: Control arm received equal number of visits but by routinely trained LHWs.	PF proportion: Lower in IG than CG than: 24.3% vs. 44.2%; RR 0.51 (95% CI [0.34, 0.78]), *P* < 0.01. EIBF proportion: Similar in IG and CG: 90.1% vs. 80.8%; RR: 1.08 (95% CI [0.97, 1.20]), *P* < 0.12. EBF proportion (at 6 months): Higher in IG than CG: 59.6% vs. 28.6%, *P* < 0.001.
Srivastava, Gupta, Bhatnagar, and Dutta (2014) India Individual RCT	Mother–baby dyads of healthy babies delivered normally at health facility.	Health facility: MNCH initiatives IG: Newborns received very early skin‐to‐skin contact (SSC; within 30 min of birth). Breastfeeding was encouraged by a nurse. CG: Standard care with no skin‐to‐skin contact. Breastfeeding was encouraged by a nurse.	EBF proportion (at 4–5 days of life): Higher in IG than CG: 86.1% vs. 66.9%, *P* = 0.002. EBF proportion (at 6 weeks): Higher in IG than CG: 85.2% vs. 63.6%, *P* < 0.001.
Talukder, Farhana, Vitta, and Greiner (2016) Bangladesh Cluster RCT	Women during pregnancy (second and third trimesters) and 6 months postpartum.	Home/family: Education and counselling IG: TBAs and community volunteers (CVs) field supervisors (FSs) received training on breastfeeding counselling for 5 days. TBAs and CVs visited women in 2nd and/or 3rd trimester of pregnancy and mothers of children aged <6 months at irregular intervals, to promote EIBF, APF, and EBF. Women were randomized to either • Group A: received support from trained TBAs/CVs • Group B: received support from trained TBAs/CVs who were supervised weekly CG: No training provided to TBAs or CVs.	EIBF proportion: Increased from baseline to end line in both IG groups; at end line significant difference between groups: IG‐A 60% vs. CG 29%, *P* < 0.05 IG‐B: 68% vs. CG 29%, *P* < 0.05 APF proportion (within 3 days of birth): Increased from baseline to end line in both IG groups; at end line significant difference between groups: IG‐A: 80% vs. CG 49%, *P* < 0.05 IG‐B: 88% vs. CG 49%, *P* < 0.05 EBF proportion: Increased from baseline to end line in both IG groups; at end line no significant difference between groups: IG‐A: 76% vs. CG 67%, *P* > 0.05 IG‐B: 83% vs. CG 67%, *P* > 0.05
Taneja et al. (2015) India Cluster RCT	HW and CHW; Mothers of newborns.	Health facility, community, home/family: MNCH initiatives IG: Integrated Management of Neonatal and Childhood Illness (IMNCI) intervention, which included • Postnatal home visits during newborn period: trained CHWs conducted home visits and counselled mothers on essential newborn practices. • Trained staff in improving health worker skills for case management of neonatal and child illness. • Health system strengthening to implement IMCI including improved supervision of CHWs and performance‐based incentives. Three monthly women's group meetings were conducted to improve community awareness of available services. CG: Routine care provided by AWW, ASHA, ANW, or primary health care physician.	EIBF proportion: The intervention resulted in a larger effect on EIBF in poorer families (difference in inequity gradients 3.0%, CI [1.5, 4.5], *P* < 0.001), in lower caste and minorities families, and in infants of mothers with fewer years of schooling.
Thakur et al. (2012) Bangladesh Individual RCT	Mothers with low birth weight babies (<2,500 g).	Health facility: Education and counselling IG: Facility‐based nutrition education on EBF was provided twice a month for 2 months after delivery. Education emphasized APF, EIBF, breast attachment, positioning, EBF for 6 months, increasing frequency and quality of maternal diet during lactation, food hygiene, personal hygiene, and necessary family assistance for breastfeeding. CG: No nutrition education.	EIBF proportion: Higher in IG than CG: 59.8% vs. 37.0%, *P* < 0.001. EBF proportion (after 2 month): Higher in IG than CG: 59.8% vs. 37.0%, *P* < 0.003.
Varghese, Roy, Saha, and Roalkvam (2014) India Quasi‐experimental (nonequivalent control group design)	Birth companions; Women during and immediately after delivery.	Health facility: MNCH initiatives IG: Yashoda programme including a facility‐based support worker or birth companion (*Yashoda*) to support mother and newborn and assist nurse in nonclinical activities. *Yashoda* supported mothers to initiated breastfeed within 1 hr of birth and counselled mothers on EBF. CG: No Yashodas were implemented.	EIBF proportion in Rajasthan: Similar in IG and CG: 41% vs. 39%; OR: 1.08 (95% CI [0.78, 1.49]). EIBF proportion in Odisha: Similar in IG and CG: 78% vs. 73%; OR: 1.09 (95% CI [0.73, 1.63]). EIBF proportion (within 5 hr) among women who delivered by caesarian section: Higher in IG than CG: 76% vs. 44%, *P* < 0.001.
Vir (2013) India Programme evaluation (before and after with control)	Pregnant women and women with infants <6 months.	Home/family: Education and counselling IG: Interpersonal communication delivered to woman and family through home visits. Maternal and child care and feeding practices counselling conducted by community‐based mobilizers *(Bal Parivar Mitras*, BPMs), volunteers selected to identify ‘at‐risk’ families. BPMs received 3‐day intensive preservice training with emphasis on selected messages and their rationale, as well as counselling skills. Pictorial counselling and monitoring family cards were used. Visits promoted child care and feeding practices. CG: Baseline survey.	EIBF proportion: Increased from 4.6% to 21.9% between baseline and end line, *P* < 0.001.
Vir, Kalita, Mondal, and Malik (2014) India Quasi‐experimental (nonequivalent control group design)	Pregnant women and their families.	Community: Community mobilization Home/family: Education and counselling IG: Nutrition security innovation (NSI) project of the Mitanin programme (community‐based peer counsellors) facilitated family‐level counselling and mobilized the community to improve coverage of maternal and child health services under the NSI project that included promotion of appropriate infant feeding. CG: State data from 1998 and 2005.	EIBF proportion: Higher in IG and CG: 52.0% vs. 44.6%, *P* < 0.05. EBF proportion (at 4–5 months): Similar in IG and CG: 80.3% vs. 73.8%, *P* > 0.05.

*Note*. CI: confidence interval; EIBF: early initiation of breastfeeding; OR: odds ratio; MNCH: maternal, newborn child health; PF: prelacteal feeds.

a Standard (WHO) indicator definitions applied, unless otherwise indicated.

Included studies were characterized by the implementation environment (health facility, community, and home/family) and type of intervention (education and counselling, community mobilization, mass media, and MNCH initiatives; Table S3). Several studies examined multiple implementation environments and intervention types. The results are presented by implementation environment and type of intervention.

### 3.2 Early initiation of breastfeeding

Twenty‐five studies reported EIBF practices, of which 21 studies showed a positive effect. The most common intervention type was interpersonal education and counselling (*n* = 18), followed by MNCH initiatives (*n* = 7), community mobilization (*n* = 4), and mass media (*n* = 1). These interventions were implemented in the home/family (*n* = 12), community (*n* = 11), and the health facility (*n* = 9) environments.

Education and counselling in the home/family environment was effective in improving EIBF outcomes in Bangladesh (Haider et al., 2000; Talukder et al., 2016) and India (Vir, 2013), and in combination with community mobilization in India (Vir et al., 2014) and Pakistan (Bhutta et al., 2008). However, a cognitive‐behavioural counselling intervention offered to pregnant women during home visits in Pakistan was not effective in improving EIBF (Sikander et al., 2015); although women received a total of seven visits, only one session was delivered before birth and one session was delivered immediately after delivery (Sikander et al., 2015). A study in Nepal reported no effect on EIBF after a sanitation and maternal and child care education intervention was delivered to women and other household members at home for 3 hr fortnightly for 6 months (Jha et al., 2006). The same study found no significant findings for six other behavioural outcomes, except care during pregnancy and introduction of complementary foods, suggesting the message content may have been too broad (Jha et al., 2006).

Community‐level education and counselling showed mixed results on EIBF proportions. In India, a women's group programme that provided community education and counselling fortnightly for 6 months with community mobilization activities had no effect on EIBF (More et al., 2012). Poor coverage, non‐specific target groups and membership attrition were characteristic of the programme and may have contributed to this finding (More et al., 2012). On the other hand, positive effects on EIBF were reported by community‐level education and counselling interventions in Bangladesh (Fottrell et al., 2013) and India (Agrawal et al., 2012; Balakrishnan et al., 2016), and when combined with community mobilization in India (Bhandari et al., 2003). All studies that examined interventions to deliver education and counselling at health facility level showed positive effect on EIBF in Bangladesh (Akter et al., 2012; Jahan et al., 2014; Thakur et al., 2012) and India (Prasad & Costello, 1995), as well. A programme in Bangladesh that combined education and counselling on EIBF at home/family level with mass media interventions also had a positive impact on EIBF (Menon et al., 2016).

MNCH initiatives, such as the Improving Maternal, Neonatal, and Child Survival programme and the Integrated Management of Neonatal and Childhood Illness (IMNCI) programme, showed positive results on EIBF. Designed to reduce neonatal and child morbidity and mortality, the programmes were implemented in a range of environments including the home/family in Bangladesh (Rahman et al., 2016), the home/family, and health facility in Pakistan (Memon et al., 2015) and at the community in Nepal (Khanal et al., 2009; Memon et al., 2015). One IMNCI intervention in India that was delivered across all three implementation environments reported a higher increase in EIBF in intervention clusters among poorer families, lower caste and minority families, and among infants of mothers with fewer years of schooling (Taneja et al., 2015). Skin‐to‐skin contact (SSC) also showed positive impacts on EIBF through home/family and community‐level interventions in India (Mahmood et al., 2011) and health facility‐level interventions in Pakistan (Kumar et al., 2008; Mahmood et al., 2011). In India, both the Janani Suraksha Yojana (JSY) conditional cash transfer programme and Yashoda birth companion programme promoted maternal and newborn health through health systems changes (Carvalho et al., 2014; Varghese et al., 2014). Though JSY showed a positive effect on EIBF proportions (Carvalho et al., 2014), the Yashoda programme showed no overall effect on EIBF except EIBF within 5 hr among infants delivered via caesarean section (Varghese et al., 2014).

### 3.3 Prelacteal feeding (APF)

All 10 studies in Bangladesh, India, and Pakistan that examined APF (or PF) reported a positive impact of the programme intervention. The most common intervention type was interpersonal education and counselling (*n* = 7), followed by MNCH initiatives (*n* = 3), community mobilization (*n* = 1), and mass media (*n* = 1). These interventions were delivered in the home/family (*n* = 5), community (*n* = 4), and health facility (*n* = 3) environments.

Education and counselling interventions delivered at home/family level improved APF in in Bangladesh (Akter et al., 2012; Haider et al., 2000; Menon et al., 2016; Talukder et al., 2016) and Pakistan (Sikander et al., 2015). Only one study in India reported positive effects from community‐level education and counselling (Bhandari et al., 2003). Two studies, one in Bangladesh (Akter et al., 2012) and the other in India (Prasad & Costello, 1995), reported that education and counselling in the health facility setting had positive effects on APF. Only two studies reported positive effects of community mobilization (Bhandari et al., 2003, India) and mass media interventions (Menon et al., 2016, Bangladesh) on APF.

MNCH initiatives that promoted SSC and kangaroo mother care (KMC) in the home/family setting in India (Kumar et al., 2008), the community in Bangladesh and India (Ahmed et al., 2011; Kumar et al., 2008), and health facilities in India (Srivastava et al., 2014) all impacted positively on APF.

### 3.4 Exclusive breastfeeding

In total, 19 studies reported effectiveness to support EBF, and all but four studies showed a positive effect. The most common intervention type was interpersonal education and counselling (*n* = 14), followed by MNCH initiatives (*n* = 6), community mobilization (*n* = 3), and mass media (*n* = 1). These interventions were delivered in the home/family (*n* = 9), community (*n* = 7), and health facility (n = 9) environments.

In India, community‐level education counselling through women's groups combined with community mobilization that addressed several health and nutrition issues showed no effect on breastfeeding practices (neither EIBF nor EBF at 4 weeks; More et al., 2012; Vir et al., 2014). A programme that used community volunteers to conduct family‐level counselling and community mobilization also reported no impact on EBF at 4–5 months (More et al., 2012, Vir et al., 2014). There was no effect on EBF of training community volunteers and traditional birth attendants to deliver education and counselling services to women at home in Bangladesh; however, the number of visits following delivery was irregular (Talukder et al., 2016). Other studies reported on the positive effects of education and counselling at home/family level on EBF in Bangladesh and Pakistan (Haider et al., 2000; Khan et al., 2016; Sikander et al., 2015) and when combined with mass media in Bangladesh (Menon et al., 2016) or community mobilization in Pakistan (Bhutta et al., 2008). Community‐level education and counselling was also effective in Bangladesh (Fottrell et al., 2013) and when combined with community mobilization in India (Bhandari et al., 2003).

All studies that examined education and counselling delivered in health facilities had a positive effect on EBF in Bangladesh (Akter et al., 2012; Jahan et al., 2014; Thakur et al., 2012) and India (Srivastava et al., 2014). Other CHW training interventions ranging from 3 days to 15 months had positive effects on EBF at 3–6 months (Bhandari et al., 2003). Similarly, mass media and community mobilization efforts reported positive EBF results (Bhandari et al., 2003; Bhutta et al., 2008; Menon et al., 2016; Vir, 2013; Vir et al., 2014).

MNCH initiatives, including SSC and KMC interventions, also showed mixed results. One KMC intervention in India encouraged mothers of very low birth weight infants to initiate KMC immediately after delivery; the intervention reported positive EIBF findings, yet there was no impact on EBF at 6 months (Gavhane et al., 2016). The JSY programme in India showed positive effects on child health outcomes including EIBF, but no effect on EBF (Carvalho et al., 2014). The authors speculate that CHWs may have provided more counselling on topics such as immunizations rather than breastfeeding (Carvalho et al., 2014). On the other hand, an SCC intervention at facility level in India reported a positive and significant effect on EBF at 6 weeks among healthy infants (Srivastava et al., 2014) as did other studies providing IMNCI packages at home/family and health facility level in India (Arifeen et al., 2009; Mazumder et al., 2014) and community and health facility level in Bangladesh (Arifeen et al., 2009, Mazumder et al., 2014).

### 3.5 Continued breastfeeding

One study in India reported CBF practices: an IMNCI intervention package delivered by CHWs to breastfeeding mothers at home, combined with CHW supervision, found no significant effect on CBF at 1 year of age (Mazumder et al., 2014).

## 4 DISCUSSION

This review synthesizes evidence from 31 studies on the effectiveness of interventions to improve breastfeeding outcomes, including EIBF, APF, EBF, and CBF, in South Asia between 1990 and 2015. The key findings from this review were that single and combination breastfeeding interventions delivered within or across multiple implementation environments (health facility, community, and home/family) improved breastfeeding practices. The findings increase our understanding of what has worked thus far, and highlight certain gaps in the literature critical for the design of future interventions and programmes to improve breastfeeding practices in the region.

Education and counselling interventions delivered to women during pregnancy and lactation in either individual or group settings improved breastfeeding practices. Strategies used included health worker training, use of interactive job aids, increased frequency, and/or duration of health worker–client interactions, as well as engagement of family and community members in group counselling sessions. These findings support existing research that demonstrates the effective use of education and counselling services to promote optimal breastfeeding practices (Haroon et al., 2013; Sinha et al., 2015). Expanding programmatic reach of these interventions through community mobilization and mass media interventions also demonstrated effectiveness to promote optimal breastfeeding practices; however, these community‐based strategies were not implemented as often as other intervention types. The findings highlight key components of successful interventions and suggest the need for more community‐level interventions to maximize the benefits education and counselling interventions.

MNCH initiatives were effective at supporting EIBF and APF but had mixed results for EBF. Most of these initiatives, including promotion of KMC and SSC, offered breastfeeding support immediately following delivery and during the postnatal period; as such, these initiatives had the most opportunity to impact on early feeding behaviours. These findings support existing guidelines supporting breastfeeding in health facilities (WHO, 2017). However, implementation of Baby Friendly Hospital Initiative (BFHI) varies in South Asia limiting the reach of interventions (Gupta & Suri, 2016). Increasing implementation and monitoring of BFHI and related MNCH interventions is a priority for action to expand coverage of effective breastfeeding interventions.

Among studies that reported no impact or negative effects of interventions on breastfeeding practices, several common characteristics were identified. These included inadequate or irregular frequency and/or duration of contacts for counselling visits and poor timing of intervention delivery relative to the mother's gestational period and the child's age to ensure breastfeeding practices are implemented early (EIBF and APF) and maintained for the recommended periods (EBF and CBF). In addition, poor coverage and targeting of interventions to pregnant and breastfeeding women, particularly for community mobilization programmes, and intervention packages that lacked context‐specific messages or covered too many health and nutrition behaviours contributed to suboptimal outcomes. The findings illustrate weaknesses in critical factors of intervention design and implementation and suggest that addressing these factors may increase the effectiveness of breastfeeding interventions in the region.

Several key knowledge gaps were identified in this review. The lack of evidence from Afghanistan is noteworthy as recent trends in Afghanistan show declining proportions in optimal breastfeeding practices (Benedict et al., 2018). There were also few studies conducted in Nepal and Pakistan, and limited evidence on APF and CBF outcomes across the South Asia region. In addition, there were no studies that examined interventions delivered in the policy or workplace environments. Additional research in these countries and on these specific breastfeeding practices and implementation environments is required to inform regional policy and programme recommendations. Furthermore, there are knowledge gaps on how interventions improved breastfeeding practices; more research and documentation on implementation pathways will contribute evidence to design more effective and scalable interventions and programmes.

## 5 LIMITATIONS

In line with scoping review methodology, our review did not grade the quality of evidence but instead described knowledge on the effectiveness of interventions to strengthen breastfeeding practices in South Asia. In order to provide the highest level of evidence on intervention effectiveness, we limited our eligibility criteria to include only randomized controlled trials and quasi‐experimental studies as these designs provide the most precise estimates of the likely effects of an intervention with limited risk of bias.

## 6 CONCLUSION

Mothers require active support to establish and sustain appropriate breastfeeding practices from birth until a child's second birthday. This scoping review of evidence from South Asia highlights that education and counselling, community mobilization, mass media, and maternal and newborn health initiatives are largely effective in improving breastfeeding practices when delivered to women and other family members at health facilities, in the community and in the family/home environment. Recommendations for the design of effective policies, programmes, and interventions emerge from this review. First, multiple intervention types and multiple intervention environments should be utilized to increase the opportunities to reach women and other family members and reinforce the promotion and support of breastfeeding. Second, the coverage, timing, frequency, duration, and targeting of intervention delivery influence effectiveness and should be carefully considered in the design of programme interventions. Notable evidence gaps were identified for specific countries (Afghanistan, Nepal, and Pakistan), breastfeeding practices (APF and CBF), and implementation environments (policy and workplace). Addressing these gaps will generate additional evidence on effective interventions and pathways to protect, promote, and support optimal breastfeeding practices in South Asia.

## CONFLICTS OF INTEREST

The authors declare that they have no conflicts of interest.

## CONTRIBUTIONS

RKB and HCC conducted analyses. RKB, HCC, RJS, and HT conceptualized the manuscript. The manuscript was written by RKB and HCC and edited by RKB, HCC, RJS, and HT.

## Supporting information

Data S1. Supporting information

## References

[mcn12697-bib-0001] Agrawal, P. K. , Agrawal, S. , Ahmed, S. , Darmstadt, G. L. , Williams, E. K. , Rosen, H. E. , & Baqui, A. H. (2012). Effect of knowledge of community health workers on essential newborn health care: A study from rural India. Health Policy and Planning, 27(2), 115–126. 10.1093/heapol/czr018 21385799 PMC3606030

[mcn12697-bib-0002] Ahmed, S. , Mitra, S. N. , Chowdhury, A. M. , Camacho, L. L. , Winikoff, B. , & Sloan, N. L. (2011). Community Kangaroo Mother Care: Implementation and potential for neonatal survival and health in very low‐income settings. Journal of Perinatology, 31(5), 361–367. 10.1038/jp.2010.131 21311502

[mcn12697-bib-0003] Akter, S. M. , Roy, S. K. , Thakur, S. K. , Sultana, M. , Khatun, W. , Rahman, R. , … Alam, N. (2012). Effects of third trimester counseling on pregnancy weight gain, birthweight, and breastfeeding among urban poor women in Bangladesh. Food Nutrition Bulletin, 33(3), 194–201. 10.1177/156482651203300304 23156122

[mcn12697-bib-0004] Arifeen, S. E. , Hoque, D. M. , Akter, T. , Rahman, M. , Hoque, M. E. , Begum, K. , … Black, R. E. (2009). Effect of the Integrated Management of Childhood Illness strategy on childhood mortality and nutrition in a rural area in Bangladesh: A cluster randomised trial. Lancet, 374(9687), 393–403. 10.1016/s0140-6736(09)60828-x 19647607

[mcn12697-bib-0005] Arksey, H. , & O'Malley, L. (2005). Scoping studies: Towards a methodological framework. International Journal of Social Research Methodology, 8(1), 19–32. 10.1080/1364557032000119616

[mcn12697-bib-0037] Balakrishnan, R. , Gopichandran, V. , Chaturvedi, S. , Chatterjee, R. , Mahapatra, T. , & Chaudhuri, I. (2016). Continuum of care services for maternal and child health using mobile technology ‐ a health system strengthening strategy in low and middle income countries. BMC Med Inform Decis Mak, 16, 84 10.1186/s12911-016-0326-z 27387548 PMC4937606

[mcn12697-bib-0006] Benedict, R. K. , Craig, H. C. , Torlesse, H. , & Stoltzfus, R. J. (2018). Trends and predictors of optimal breastfeeding among children 0–23 months, South Asia: Analysis of national survey data. Maternal & Child Nutrition, 14(Suppl 4), e12698 10.1111/mcn.12698 30499250 PMC6519202

[mcn12697-bib-0007] Bhandari, N. , Bahl, R. , Mazumdar, S. , Martines, J. , Black, R. E. , & Bhan, M. K. (2003). Effect of community‐based promotion of exclusive breastfeeding on diarrhoeal illness and growth: A cluster randomised controlled trial. Lancet, 361(9367), 1418–1423. 10.1016/s0140-6736(03)13134-0 12727395

[mcn12697-bib-0008] Bhutta, Z. A. , Memon, Z. A. , Soofi, S. , Salat, M. S. , Cousens, S. , & Martines, J. (2008). Implementing community‐based perinatal care: Results from a pilot study in rural Pakistan. Bulletin of the World Health Organization, 86(6), 452–459. 18568274 10.2471/BLT.07.045849 PMC2647462

[mcn12697-bib-0009] Carvalho, N. , Thacker, N. , Gupta, S. S. , & Salomon, J. A. (2014). More evidence on the impact of India's conditional cash transfer program, Janani Suraksha Yojana: quasi‐experimental evaluation of the effects on childhood immunization and other reproductive and child health outcomes. PLoS One, 9(10), e109311 10.1371/journal.pone.0109311 25303078 PMC4193776

[mcn12697-bib-0010] Dibley, M. J. , Roy, S. K. , Senarath, U. , Patel, A. , Tiwari, K. , Agho, K. E. , & Mihrshahi, S. (2010). Across‐country comparisons of selected infant and young child feeding indicators and associated factors in four South Asian countries. Food and Nutrition Bulletin, 31(2), 366–375. 10.1177/156482651003100224 20707239

[mcn12697-bib-0038] Fottrell, E. , Azad, K. , Kuddus, A. , Younes, L. , Shaha, S. , Nahar, T. , … Houweling, T.A. (2013). The effect of increased coverage of participatory women's groups on neonatal mortality in Bangladesh: A cluster randomized trial. JAMA Pediatr, 167(9), 816–825. 23689475 10.1001/jamapediatrics.2013.2534 PMC5082727

[mcn12697-bib-0011] Gavhane, S. , Eklare, D. , & Mohamm, H. (2016). Long term outcomes of Kangaroo Mother care in very low birth weight infants. Journal of Clinical and Diagnostic Research, 10(12), SC13–SC15. 10.7860/JCDR/2016/23855.9006 PMC5296538 28208965

[mcn12697-bib-0012] Gupta, A. , & Suri, S. (2016). Has your nation done enough to bridge the gaps? World breastfeeding trends initiative (WBTi).

[mcn12697-bib-0013] Haider, R. , Ashworth, A. , Kabir, I. , & Huttly, S. R. (2000). Effect of community‐based peer counsellors on exclusive breastfeeding practices in Dhaka, Bangladesh: A randomised controlled trial. Lancet, 356(9242), 1643–1647. 10.1016/S0140-6736(00)03159-7 11089824

[mcn12697-bib-0014] Haroon, S. , Das, J. K. , Salam, R. A. , Imdad, A. , & Bhutta, Z. A. (2013). Breastfeeding promotion interventions and breastfeeding practices: A systematic review. BMC Public Health, 13(Suppl 3), S20 10.1186/1471-2458-13-S3-S20 24564836 PMC3847366

[mcn12697-bib-0039] Jahan, K. , Roy, S. K. , Mihrshahi, S. , Sultana, N. , Khatoon, S. , Roy, H. , … Steele, J. (2014). Short-term nutrition education reduces low birthweight and improves pregnancy outcomes among urban poor women in Bangladesh. Food Nutr Bull, 35(4), 414–421. 25639126 10.1177/156482651403500403

[mcn12697-bib-0015] Jha, N. , Kumar, S. , Yadav, B. K. , Singh, G. C. , & Niraula, S. R. (2006). Impact of family health exercise program on health knowledge and practice of a rural population of eastern Nepal. Kathmandu University Medical Journal (KUMJ), 4(1), 44–47. 18603867

[mcn12697-bib-0040] Khan, A. I. , Kabir, I. , Eneroth, H. , El Arifeen, S. , Ekstrom, E. C. , Frongilo, E. A. , & Persson, L. A. (2016). Effect of a randomised exclusive breastfeeding counselling intervention nested into the MINIMat prenatal nutrition trial in Bangladesh. Acta Paediatr. 10.1111/apa.13601 PMC5215617 27659772

[mcn12697-bib-0016] Khanal, S. , Zhang, W. D. , & Khanal, S. (2009). The efficacy of community based intervention in newborn care practices and neonatal illness management in Morang District of Nepal. Life Science Journal, 7(4), 60–67.

[mcn12697-bib-0017] Kumar, V. , Mohanty, S. , Kumar, A. , Misra, R. P. , Santosham, M. , Awasthi, S. , … Darmstadt, G. L. (2008). Effect of community‐based behaviour change management on neonatal mortality in Shivgarh, Uttar Pradesh, India: A cluster‐randomised controlled trial. Lancet, 372(9644), 1151–1162. 10.1016/s0140-6736(08)61483-x 18926277

[mcn12697-bib-0018] Mahmood, I. , Jamal, M. , & Khan, N. (2011). Effect of mother‐infant early skin‐to‐skin contact on breastfeeding status: A randomized controlled trial. Journal of the College of Physicians and Surgeons Pakistan, 21(10), 601–605. 10.2011/jcpsp.601605 22015120

[mcn12697-bib-0019] Mazumder, S. , Taneja, S. , Bahl, R. , Mohan, P. , Strand, T. A. , Sommerfelt, H. , … Bhandari, N. (2014). Effect of implementation of integrated management of neonatal and childhood illness programme on treatment seeking practices for morbidities in infants: Cluster randomised trial. The BMJ, 349, g4988 10.1136/bmj.g4988 25172514 PMC4148946

[mcn12697-bib-0020] Memon, Z. A. , Khan, G. N. , Soofi, S. B. , Baig, I. Y. , & Bhutta, Z. A. (2015). Impact of a community‐based perinatal and newborn preventive care package on perinatal and neonatal mortality in a remote mountainous district in Northern Pakistan. BMC Pregnancy and Childbirth, 15, 106 10.1186/s12884-015-0538-8 25925407 PMC4446857

[mcn12697-bib-0021] Menon, P. , Nguyen, P. H. , Saha, K. K. , Khaled, A. , Kennedy, A. , Tran, L. M. , … Rawat, R. (2016). Impacts on breastfeeding practices of at‐scale strategies that combine intensive interpersonal counseling, mass media, and community mobilization: Results of cluster‐randomized program evaluations in Bangladesh and Viet Nam. PLoS Medicine, 13(10), e1002159 10.1371/journal.pmed.1002159 27780198 PMC5079648

[mcn12697-bib-0022] More, N. S. , Bapat, U. , Das, S. , Alcock, G. , Patil, S. , Porel, M. , … Osrin, D. (2012). Community mobilization in Mumbai slums to improve perinatal care and outcomes: A cluster randomized controlled trial. PLoS Medicine, 9(7), e1001257 10.1371/journal.pmed.1001257 22802737 PMC3389036

[mcn12697-bib-0042] Prasad, B. , & Costello, A. M. (1995). Impact and sustainability of a “baby friendly” health education intervention at a district hospital in Bihar, India. BMJ, 310(6980), 621–623. 7703747 10.1136/bmj.310.6980.621 PMC2549005

[mcn12697-bib-0023] Rahman, M. , Yunus, F. M. , Shah, R. , Jhohura, F. T. , Mistry, S. K. , Quayyum, T. , … Afsana, K. (2016). A controlled before‐and‐after perspective on the improving maternal, neonatal, and child survival program in rural Bangladesh: An impact analysis. PLoS One, 11(9), e0161647 10.1371/journal.pone.0161647 27583478 PMC5008808

[mcn12697-bib-0024] Sikander, S. , Maselko, J. , Zafar, S. , Haq, Z. , Ahmad, I. , Ahmad, M. , … Rahman, A. (2015). Cognitive‐behavioral counseling for exclusive breastfeeding in rural pediatrics: A cluster RCT. Pediatrics, 135(2), e424–e431. 10.1542/peds.2014-1628 25583916

[mcn12697-bib-0025] Sinha, B. , Chowdhury, R. , Sankar, M. J. , Martines, J. , Taneja, S. , Mazumder, S. , … Bhandari, N. (2015). Interventions to improve breastfeeding outcomes: A systematic review and meta‐analysis. Acta Paediatra, 104(467), 114–134. 10.1111/apa.13127 26183031

[mcn12697-bib-0026] Srivastava, S. , Gupta, A. , Bhatnagar, A. , & Dutta, S. (2014). Effect of very early skin to skin contact on success at breastfeeding and preventing early hypothermia in neonates. Indian Journal of Public Health, 58(1), 22–26. 10.4103/0019-557x.128160 24748353

[mcn12697-bib-0027] Talukder, S. , Farhana, D. , Vitta, B. , & Greiner, T. (2016). In a rural area of Bangladesh, traditional birth attendant training improved early infant feeding practices: A pragmatic cluster randomized trial. Maternal & Child Nutrition, 13 10.1111/mcn.12237 PMC6865915 26775711

[mcn12697-bib-0028] Taneja, S. , Bahl, S. , Mazumder, S. , Martines, J. , Bhandari, N. , & Bhan, M. K. (2015). Impact on inequities in health indicators: Effect of implementing the integrated management of neonatal and childhood illness programme in Haryana, India. Journal of Global Health, 5(1), 010401 10.7189/jogh.05.010401 25674350 PMC4306296

[mcn12697-bib-0041] Thakur, S. K. , Roy, S. K. , Paul, K. , Khanam, M. , Khatun, W. , & Sarker, D. (2012). Effect of nutrition education on exclusive breastfeeding for nutritional outcome of low birth weight babies. European Journal of Clinical Nutrition, 66(3), 376–381. https://doi.org/10.1038/ejcn.2011.182 22085870 10.1038/ejcn.2011.182

[mcn12697-bib-0029] UNICEF (2014). State of the World's children 2015. New York, NY: UNICEF.

[mcn12697-bib-0030] UNICEF (2016). From the first hour of life: Making the case for improved infant and young child feeding everywhere. New York, NY: United Nations Children's Fund (UNICEF).

[mcn12697-bib-0031] Varghese, B. , Roy, R. , Saha, S. , & Roalkvam, S. (2014). Fostering maternal and newborn care in India the Yashoda way: Does this improve maternal and newborn care practices during institutional delivery? PLoS One, 9(1), e84145 10.1371/journal.pone.0084145 24454718 PMC3893122

[mcn12697-bib-0032] Victora, C. G. , Bahl, R. , Barros, A. J. , Franca, G. V. , Horton, S. , Krasevec, J. , … Grp Lancet Breastfeeding Series (2016). Breastfeeding in the 21st century: Epidemiology, mechanisms, and lifelong effect. Lancet, 387(10017), 475–490. 10.1016/S0140-6736(15)01024-7 26869575

[mcn12697-bib-0033] Vir, S. C. (2013). Community based maternal and child health nutrition project, uttar pradesh: An innovative strategy focusing on "at risk" families. Indian Journal of Community Medicine, 38(4), 234–239. 10.4103/0970-0218.120159 24302825 PMC3831694

[mcn12697-bib-0034] Vir, S. C. , Kalita, A. , Mondal, S. , & Malik, R. (2014). Impact of community‐based mitanin programme on undernutrition in rural Chhattisgarh State, India. Food and Nutrition Bulletin, 35(1), 83–91. 10.1177/156482651403500110 24791582

[mcn12697-bib-0035] WHO (2003). Global strategy for infant and young child feeding. Geneva: World Health Organization (WHO)/United Nations Children's Fund (UNICEF).

[mcn12697-bib-0036] WHO (2017). Guideline: Protecting, promoting and supporting breastfeeding in facilities providing maternity and newborn services. Geneva: World Health Organization. 29565522

