# Supplementary material for: Effectiveness of programmes and interventions to support optimal breastfeeding among children 0–23 months, South Asia: A scoping review
Source: Matern Child Nutr. 2018 Nov 29;14(Suppl 4):e12697. doi: 10.1111/mcn.12697 (PMC6519148; doi:10.1111/mcn.12697)
Supplement: Supplementary file 1 — Data S1. Supporting information [file MCN-14-e12697-s001.docx]

# **SUPPLEMENTARY APPENDIX**

## Appendix 1.

| Afghan* or Bangladesh* or India* or Nepal* or Pakistan* or “South Asia*" or “southern asia*”  Promot* or support* or protect* or improv* or effect* or impact or campaign or program or polic* or scheme or service or approach or interven* or guid* or advice or advise or counsel*  Breastfe* or “breast fe*” or breastmilk or “breast milk” or “human milk” or “maternal milk” or pre-lacteal or “pre lacteal” or prelacteal or lactati* or colostrum  initiat* or early or delay or timely or exclusive* or continu* or partial* or prolong* or rate or practice* |
| --- |

Figure A1. Example of search terms.

## Appendix 2.

Records identified through contacting experts (n=21)

Studies eligible after full-text review

(n=2)

Full-text not accessible

(n=16)

Studies eligible after full-text review

(n=29)

Full-text screened for eligibility

(n=231)

Full-text excluded

(n=221)

Duplicates removed

(n=303)

Records identified through database searching

(n=1439)

Records screened

(n=1136)

Excluded after review of title/abstract

(n=889)

Included studies (n=31)

Figure A2. Flow chart of the study selection process for eligible studies.

## Appendix 3.

## Table A3. Studies that report evidence on effectiveness to improve breastfeeding practices by intervention characteristics (N=31).

| **Breastfeeding practice** | **Level of implementation** | **Intervention type** | **Bangladesh**  **(n=11)** | **India**  **(n=14)** | **Nepal**  **(n=2)** | **Pakistan**  **(n=4)** |
| --- | --- | --- | --- | --- | --- | --- |
| **EIBF (n=25)** | **Home/family environment (n=12)** | Education and counseling | (+) Haider 2000  (+) Menon 2016^b,c^  (+) Talukder 2016^a^ | (+) Vir 2013  (+) Vir 2014^a,b,c^ | (-) Jha 2006 | (+) Bhutta 2008^b,c^  (-) Sikander 2015^a^ |
|  |  | MNCH initiatives | (+) Rahman 2016 | (+) Kumar 2008^b^  (+) Taneja 2015^b^ | *none* | (+) Memon 2015^b^ |
|  | **Community environment (n=11)** | Community mobilization | *none* | (+) Bhandari 2003^c^  (-) More 2012^c^  (+) Vir 2014^a,b,c^ | *none* | (+) Bhutta 2008^b,c^ |
|  |  | Education and counseling | (+) Menon 2016^b,c^  (+) Fottrell 2013 | (+) Agrawal 2012  (+) Balakrishnan 2016  (+) Bhandari 2003^c^  (-) More 2012^c^ | *none* | *none* |
|  |  | Mass media | (+) Menon 2016^2,3^ | *none* | *none* | *none* |
|  |  | MNCH initiatives | *none* | (+) Kumar 2008^b^  (+) Taneja 2015^b^ | (+) Khanal 2009 | *none* |
|  | **Health facility environment (n=9)** | Education and counseling | (+) Akter 2012  (+) Jahan 2008  (+) Thakur 2012 | (+) Prasad 1995 | *none* | *none* |
|  |  | MNCH initiatives | *none* | (+) Carvalho 2014^a^  (+) Taneja 2015^b^  (-) Varghese 2014 | *none* | (+) Mahmood 2011  (+) Memon 2015^b^ |
| **APF/PF (n=10)** | **Home/family environment (n=5)** | Education and counseling | (+) Haider 2000  (+) Menon 2016^b,c^  (+) Talukder 2016^a^ | *none* | *none* | (+) Sikander 2015^a^ |
|  |  | MNCH initiatives | *none* | (+) Kumar 2008^b^ | *none* | *none* |
|  | **Community environment (n=4)** | Community mobilization | *none* | (+) Bhandari 2003^c^ | *none* | *none* |
|  |  | Education and counseling | *none* | (+) Bhandari 2003^c^ | *none* | *none* |
|  |  | Mass media | (+) Menon 2016^b,c^ | *None* | *none* | *none* |
|  |  | MNCH initiatives | (+) Ahmed 2011 | (+) Kumar 2008^b^ | *none* | *none* |
|  | **Health facility environment (n=3)** | Education and counseling | (+) Akter 2012 | (+) Prasad 1995 | *none* | *none* |
|  |  | MNCH initiatives |  | (+) Srivastava 2014 |  |  |
| **EBF (n=19)** | **Home/family environment (n=9)** | Education and counseling | (+) Haider 2000  (+) Khan 2016  (+) Menon 2016^b,c^  (-) Talukder 2016^a^ | (-) Vir 2014^a,b,c^ | *none* | (+) Bhutta 2008^b,c^  (+) Sikander 2015^a^ |
|  |  | MNCH initiatives | *none* | (+) Mazumder 2014 | *none* | (+) Mahmood 2011 |
|  | **Community environment (n=7)** | Community mobilization | *none* | (+) Bhandari 2003^c^  (-) More 2012^c^ | *none* | (+) Bhutta 2008^b,c^ |
|  |  | Education and counseling | (+) Fottrell 2013 | (+) Bhandari 2003^c^  (-) More 2012^c^  (-) Vir 2014^a,b,c^ | *none* | *none* |
|  |  | Mass media | (+) Menon 2016^b,c^ | *none* | *none* | *none* |
|  |  | MNCH initiatives | (+) Arifeen 2009^2^ | *none* | *none* | *none* |
|  | **Health facility environment (n=9)** | Education and counseling | (+) Akter 2012  (+) Jahan 2008  (+) Thakur 2012 | (+) Srivastava 2014 | *none* |  |
|  |  | MNCH initiatives | (+) Arifeen 2009^b^ | (-) Carvalho 2014^a^  (-) Gavhane 2016  (+) Mazumder 2014^b^  (+) Srivastava 2014 | *none* | *none* |
| **CBF (n=1)** | **Home/family environment (n=1)** | MNCH initiatives | *none* | (+) Mazumder 2014^b^ | *none* | *none* |

(+) Denotes a positive impact on breastfeeding practices. (-) Denotes a negative impact or no effect on breastfeeding practices.

^a^ Study reports positive effectiveness for one breastfeeding practice and no effectiveness for another breastfeeding practice (Carvalho et al., 2014; Sikander et al., 2015; Talukder et al., 2016; Vir et al., 2014).

^b^ Study examines programs or approaches implemented across multiple levels of implementation (Arifeen et al., 2009; Bhutta et al., 2008; Kumar et al., 2008; Mazumder et al., 2014; Memon et al., 2015; Menon et al., 2016; Taneja et al., 2015; Vir et al., 2014).

^c^ Study examines programs or approaches implemented through multiple intervention types (Bhandari et al., 2003; Bhutta et al., 2008; Menon et al., 2016; More et al., 2012; Vir et al., 2014)
